# Developing a Core Outcome Set for Assessing Clinical Safety Outcomes of Prostate Cancer in Clinical Trials of Traditional Chinese Medicine: Protocol for a Mixed Methods Study

**DOI:** 10.2196/46794

**Published:** 2023-08-07

**Authors:** Huichuan Tian, Yao Zhang, Jiajun Ren, Chaoran Wang, Ruiyu Mou, Xiaojiang Li, Yingjie Jia

**Affiliations:** 1 The First Affiliated Hospital of Tianjin University of Chinese Medicine Tianjin China; 2 National Clinical Research Center of Chinese Acupuncture and Moxibustion Tianjin China

**Keywords:** core outcome set, safety outcomes, prostate cancer, traditional Chinese medicine, clinical trial, study protocol, quality of life, efficacy, clinical safety outcome, men

## Abstract

**Background:**

Among the common malignant tumors in men worldwide, the incidence of prostate cancer ranks second to lung cancer. This disease will bring an economic burden to patients and their families and can reduce the quality of life of patients. Researchers have conducted numerous clinical trials on the efficacy and safety of different interventions in the treatment of prostate cancer with traditional Chinese medicine (TCM) combined with standard treatment regimens. However, the currently published clinical trials exhibit inconsistent and irregular reporting of outcome measures.

**Objective:**

The objective of this paper is to emphasize the need for a core outcome set (COS) to facilitate future prostate cancer research, aiming to improve the quality of trials and generate high-quality evidence.

**Methods:**

This mixed methods project has three phases, as follows: (1) a scoping review of the literature to identify outcomes that have been reported in clinical trials and systematic reviews of interventions involving TCM for the treatment of prostate cancer as well as a qualitative component using interviews to obtain the views of patients with prostate cancer, their families, and their caregivers who have a history of TCM treatment; (2) a Delphi survey among stakeholders to prioritize the core outcomes—Participants will include traditional Chinese and Western medicine clinicians in prostate cancer–related directions, nurses, and methodology experts who will participate in 2 rounds of the Delphi method expert consultation to score each outcome in the list of outcome indicators; and (3) a face-to-face consensus meeting to discuss and agree on the final COS for the application of TCM in the treatment of prostate cancer.

**Results:**

The protocol has been registered in PROSPERO (CRD42022356184) before the start of the review process, and we will initiate the review on August 1, 2023; results should be expected by September 1, 2023. The Delphi survey among stakeholders is expected to start in October 2023.

**Conclusions:**

The development of a core outcome set for assessing clinical safety outcomes of prostate cancer in clinical trials of TCM will provide a significant first step to assist Chinese doctors, researchers, and policy makers.

**Trial Registration:**

PROSPERO CRD42022356184; https://tinyurl.com/ysakz74r

**International Registered Report Identifier (IRRID):**

PRR1-10.2196/46794

## Introduction

Among the common malignant tumors in men worldwide, prostate cancer ranks second to lung cancer in terms of incidence, accounting for 14.1% of the total number of malignant tumors; the mortality rate for prostate cancer is 6.8%, ranking fifth in male cancer mortality, and making it the most common type of cancer in 112 countries globally [1]. According to the new cancer data in China in 2019, the incidence of prostate cancer ranks sixth among men, its mortality rate ranks ninth, and the incidence is on the rise in the country [2]. Some scholars predict that by 2030, prostate cancer will surpass gastric cancer to become the third most common cancer in men [3]. Patients with prostate cancer do not have obvious clinical symptoms in the early stages, 50%-80% of patients are diagnosed at an advanced stage [4], and approximately 80% of patients diagnosed with advanced prostate cancer may develop bone metastasis [5]. Currently, follow-up observation, surgical treatment, endocrine therapy, chemotherapy, external radiation therapy, and seed implantation internal radiation therapy can be used for prostate cancer treatment. Among these options, endocrine therapy—represented by Androgen Deprivation Therapy (ADT)—has become an internationally recognized standard treatment for advanced prostate cancer. The advantage of ADT is that it can significantly delay the progression of the tumor, but the treatment period is not long, usually lasting only 1-2 years. If the treatment time exceeds 2 years, the body will easily develop resistance to ADT, and almost all of patients will progress to castration-resistant prostate cancer in later stages or metastatic castration-resistant prostate cancer [6]. Once the disease progresses to castration-resistant prostate cancer, radiotherapy, chemotherapy, targeted therapy, and other methods are difficult to effectively slow down the progress of the disease; ADT usually produces many adverse reactions [7], reducing the quality of life of patients during treatment [8]. Therefore, modern medicine still has many deficiencies in the diagnosis and treatment of prostate cancer, and there is still a lack of effective treatment methods.

In recent years, with the in-depth understanding of traditional Chinese medicine (TCM) regarding the etiology, pathogenesis, and treatment of prostate cancer, clinical research on TCM for the treatment of prostate cancer at various stages has gradually increased [9,10]. Numerous clinical trials and systematic reviews have been conducted on TCM or integrative medicine for prostate cancer to improve the efficacy and safety outcomes [11-14]. However, due to the lack of guidance on a core outcome set (COS), there is obvious heterogeneity in the selection of outcome measures among different studies. Meanwhile, TCM treatment includes a variety of interventions, such as Chinese medicine decoction, Chinese medicine capsule, Chinese medicine injection, and topical Chinese medicine patch therapy [15,16]. Hence, the complexity and diversity of clinical trials of TCM for prostate cancer makes it even more important to evaluate the efficacy of different interventions. How to select high-quality outcome indicators based on clinical trials is an urgent problem to be solved. A point that cannot be ignored is that the inconsistent and nonstandard reporting of outcome indicators and the status of adverse events is often underestimated or ignored, which may lead to a waste of medical resources, a reduction in clinical treatment efficacy, and an impact on the foundation of clinical trials and evidence-based clinical decision-making.

Therefore, to facilitate future research on TCM treatment of prostate cancer, we need to develop a COS. COS, as an agreed-upon set of standard outcomes for a specific disease or condition, can reduce the risk of reporting bias by prompting researchers to focus their reporting on a more specific set of outcomes that stakeholders agree are best suited to their field [17-19]. There is also the potential to reduce economic waste; improve the utility of randomized controlled trials; facilitate comparison of treatments across different sources of evidence; and aid in the development of systematic reviews, meta-analyses, and evidence-based clinical guidelines through standardized reporting of results in specific areas [20].

## Methods

### Methods Overview

**Figure 1:**
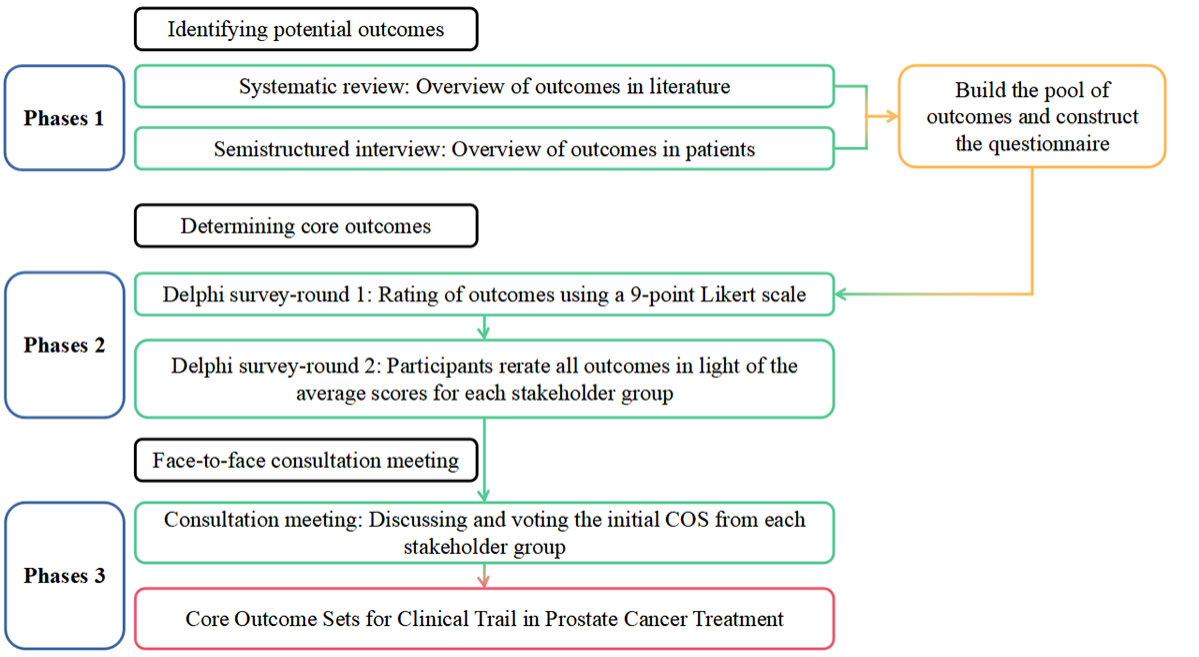
Overview of the core outcome set (COS) development process.

This COS initiative was registered in January 2022 with the Core Outcome Measures in Effectiveness trial (COMET) initiative. We followed the Core Outcome Set-STAndards for Development [17], the Core Outcome Set-STAndards for Reporting [18], and the Core Outcome Set-STAndardised Protocol items [21] (Multimedia Appendix 1) for developing and reporting this COS. This section describes the 4 phases of the COS development using an international, web-based Delphi study, following the guidelines for a COS development [19]. Figure 1 presents an overview of the 3 phases.

Meanwhile, we will set up a research working group who will be responsible for the planning, guidance, and supervision of the research process of this project. The research group will comprise 5 experts, including 2 TCM or integrated TCM specialists in oncology, a Western medicine specialist in oncology, a nurse, and a methodologist. The Steering Committee will review and confirm the research protocol, identify a list of preliminary results, make decisions in case of confusion, and attend consensus meetings to facilitate the development of the core indicator set.

### Ethics Approval

This study has been granted ethics approval by the First Teaching Hospital of Tianjin University of Traditional Chinese Medicine Research Ethics Committee (TYLL2022[K]-21).

### Phase 1: Identifying Potential Outcomes

We will identify potential outcomes used in studies evaluating the TCM clinical efficiency and safety in the treatment of prostate cancer by performing a systematic review. A semistructured interview will also be organized to identify outcomes that patients perceive as relevant and important (phase 1).

#### Identification of Outcomes Through a Scoping Review

A scoping review will be performed following the PRISMA (Preferred Reporting Items for Systematic Reviews and Meta-Analysis) checklist. This study protocol was registered in the PROSPERO database (CRD42022356184) before the start of the review process.

We will search 8 databases (PubMed, Embase, Cochrane, Clinical Trials, China National Knowledge Infrastructure, Wanfang Data knowledge service platform, and Chinese Scientific Journal Database), including search terms that encompass TCM and related preparations, prostate cancer, and controlled trials.

The English search strategy is presented in Multimedia Appendix 1. Studies will be included if they meet the following criteria: (1) clinical research (the type of research is not limited); (2) the research participants are patients with prostate cancer; (3) there are clear inclusion and exclusion criteria; (4) the intervention measures are Chinese medicine and Chinese medicine combined with Western medicine treatment, and the control measures are Chinese medicine and Western medicine treatment, a placebo, or blank control; (5) randomized controlled trials that have at least 30 patients in each group, and observational studies with at least 50 patients; (6) for published research, the language is limited to Chinese and English.

Data extraction included the name of the first author, number of participants, outcome measures (including primary and secondary outcomes), the definition of outcome measures, outcome measurement tools, and timing. In addition, the quality of outcome reporting and methodological quality will be assessed. After the data extraction is completed, it is necessary to standardize and merge the outcome index domain or the outcome index. Two researchers will jointly standardize and merge the outcome index domain or the outcome index and reach an agreement through negotiation.

Subsequently, 2 reviewers will independently extract relevant data from the included studies using a standardized data extraction sheet. Information will be extracted regarding the study design, study population, intervention characteristics, reported outcomes, and measurement tools. Disagreement will be resolved through discussion with a third reviewer. Results will include all unique outcomes and a frequency table of the most reported outcomes, sorted per outcome domain [22]. This list of unique outcomes will be presented to the expert panel (phase 2).

#### Identification of Outcomes Through Semistructured Interviews

It is necessary to consult patients on prostate cancer. Semistructured interviews will be conducted to ensure that certain topics are discussed, while giving the patient the flexibility to delve into details when needed. During patient interviews, information will be collected about health status, previous treatments and expectations, and inconveniences experienced during treatment. Patients aged ≥18 years who were diagnosed with prostate cancer in pathology and regularly received TCM or integrated Chinese and Western medicine treatment over 3 months and their family members or caregivers will be invited to participate in the semistructured interviews. Informed consent is required to recruit participants to this study. Patients will be assured at the start of the interview that they can choose to opt out at any time during the interview if they feel unable or uncomfortable to complete the interviews. The specific inclusion and exclusion criteria are presented in Textbox 1.

**The inclusion and exclusion criteria for the semistructured interview.**

**Inclusion criteria**

- Patients with prostate cancer (pathological diagnosis according to the national guidelines for diagnosis and treatment of prostate cancer 2022 in China [23])
- Patients ≥18 years of age
- Traditional Chinese medicine (TCM) treatment or integrated TCM and Western medicine treatment ≥3 months
- Patients who signed the informed consent forms

**Exclusion criteria**

- Patients with a severe mental disease, cancer, and other life-threatening diseases
- Primary malignancies other than prostate cancer
- Participants judged by the investigator to be inappropriate for participation
- The patient refuses to participate in the study

#### Recruitment of Interviewees

Patient interviews will be conducted in China to ensure that the representative views of patients are included. We plan to interview potential patients in inpatient wards and outpatient clinics of the First Affiliated Hospital of Tianjin University of Traditional Chinese Medicine. There is no strict standard for the sample size of semistructured interviews. To achieve data saturation [24], based on the previous research experience, more than 30 patients will be recruited to ensure obtaining comprehensive results [25,26]. We believe that this sample size will be sufficient to achieve saturation in the semistructured interviews. However, if new viewpoints emerge during the interview, the sample size of the interview will increase. Patients participating in the interviews can complete questionnaires with the help of researchers. To improve the representativeness of the sample in the sample selection process, we will select patients with different ages, disease stages, disease severity, education, occupation, and family income.

#### Data Collection and Analysis

The questions in the semistructured interviews are as follows:

1. What are the most physiological and psychological effects of prostate cancer on you?
2. What kind of treatment (eg, radical prostatectomy, radiation therapy, and androgen deprivation therapy) have you received after the prostate cancer diagnosis?
3. What therapeutic effect do you want to achieve?
4. What kind of inconvenience does the current Chinese medicine treatment bring to you?
5. Which outcomes are important to you? Which one is the most important?

The patient interviews were audio recorded. After each interview, the recording was transcribed into text and read word by word to form a thematic framework and extract relevant outcome indicators [27]. The outcome indicators were standardized by 2 investigators and compared with the original outcome indicator list formed by the systematic review. If an outcome indicator was judged to be new, it was included in the Delphi survey.

#### Pooling and Grouping of Study Outcomes

After completing the original list of outcome indicators, according to the recommendations of the “COMET Handbook” [20], the standardized and merged outcome indicators were classified to form a list of outcome indicators of clinical safety outcomes of prostate cancer in clinical trials of TCM. Specific steps for this procedure are as follows:

1. Translating English into Chinese according to the terms formulated by the National Science and Technology Terminology Committee; if there are no relevant terms, 2 researchers will determine the appropriate translation.

2. Extracting the combined results as separate results.

3. Combining overlapping results into one result according to the definition of the result (eg, death, death from any cause, mortality, and total mortality)

4. Discarding results without definition or measurement tools.

5. Classifying the outcomes into different outcome areas according to the classifications developed by the COMET initiative (two researchers will cross-validate the results).

Any inconsistencies will be discussed further by the 2 researchers until a consensus is reached. Next, the results will be formatted as questions and submitted to the Delphi survey [28].

### Phase 2: the Delphi Survey

#### Stakeholders’ Involvement

We will invite health industry stakeholders, such as prostate cancer clinicians, senior nurses, and researchers, and evidence-based medicine methodologists, to participate in 2 rounds of the Delphi survey [29]. The inclusion criteria for the participants in the Delphi survey are shown in Textbox 2. There are no exclusion criteria.

**The inclusion criteria for health professionals in the Delphi survey.**

**Inclusion criteria**

- Health professionals with a bachelor’s degree or above.
- Health professionals who have at least a senior title or above.
- Clinicians and nurses should have work experience in tertiary hospitals.
- There will be no restriction on the professionals’ geographical area.
- The researchers (ie, first author, corresponding author, or other authors) should have published at least one clinical study related to traditional Chinese medicine.

#### Sampling

There is no consensus on the optimal sample size for the Delphi study, and based on the content of published literature, sample sizes for health professionals typically range from 30 to 120 participants [22,25,26]. In this study, following the research purpose of the project, we adhered to the principles of authority, representativeness, and regionality. We invited about 50 TCM or integrated TCM clinicians, nurses, and methodological experts specializing in prostate cancer–related areas to participate in 2 rounds of the Delphi survey.

#### Round 1 of the Delphi Survey

The first round of the Delphi survey will include all candidate results and ratings in different outcome areas. The questionnaire will be on a 9-point scale [20], where “1-3” indicates that the result is not important in the COS, “4-6” indicates that the result is important but not critical in the COS, and “7-9” indicates that the result is critical in the COS. At the end of the questionnaire, there will be an open-ended question: “What results do you think are important but not included in the questionnaire?”

Data analysis for the first round of the Delphi survey will include calculating the frequency of response options for each outcome. If no more than 10% of the participants who completed the questionnaire scored an outcome measure of 7-9, that result will be excluded from the second round of the Delphi survey. If participant-recommended results were not included in the first Delphi survey, 2 researchers will determine whether they are new or overlapping with existing outcomes. The new results will be included in the second round of the Delphi survey. Questionnaires for the Delphi law experts were distributed via email or WeChat (a Chinese web-based social software) and returned within 2 weeks.

#### Data Analysis for Round 1 of the Delphi Survey

We will use Microsoft Excel and SPSS 21.0 software (IBM Corp) and include descriptive analysis of the expert’s basic information, including the expert’s title, professional field, education, and years of experience, to understand the authority of expert consultation. We will analyze the responses from the first round, calculating the response rate and frequency of response options for each outcome. Moreover, we will analyze the distribution of scores for each stakeholder group. All results will be carried over to round 2 of the Delphi survey.

#### Round 2 of the Delphi Survey

The second round of the Delphi survey will be sent to participants who completed the first round. In the questionnaire, participants will receive scores from the first round of the Delphi survey and their stakeholder score distribution. They will be asked to rescore the results within 3 weeks. We will send an email or message to the participants, reminding them to complete the Delphi survey by the end of week 2. If the response rate is <80%, we will extend the opening hours of the Delphi survey or invite other eligible individuals to participate in the survey.

#### Data Analysis for Round 2 of the Delphi Survey

Data analysis for round 2 of the Delphi survey will include response rates; frequency of responses for each outcome option; distribution of scores for each stakeholder group; the outcomes that achieve “consensus in,” “consensus out,” and “no consensus”; and attrition bias. As shown in Table 1, each outcome will be defined into 3 categories [29]. SPSS 21.0 (IBM Corp) will be used to calculate the positive coefficient, degree of authority, and coordination coefficient of participants to demonstrate the validity of the 2 rounds of the Delphi expert consultation.

Attrition bias resulting from missing data will be determined by calculating the average score for each outcome among participants who completed or did not complete the 2 Delphi survey rounds. The missing data will not be considered if there is no attrition bias. The missing data will be considered as “unclear” if there is an attrition bias or if participants did not score all safety outcomes [30,31].

**Table 1:** Definition of consensus.

Classification consensus	Criteria	Interpretation
Consensus in	≥70% of the participants rated the outcome 7-9 <15% of the participants rated the outcome 1-3 The average patient rating is ≥7, regardless of other scores	Outcome is important
Consensus out	≥70% of the participants rated the outcome 1-3 <15% of the participants rated the outcome 7-9	Outcome is not important
Consensus to be further discussed	All other results	Potentially important outcome

### Phase 3: Consensus Meeting

#### Stakeholder Selection

We will invite experts who have completed the 2 rounds of the Delphi survey to conduct a face-to-face consensus meeting to discuss and vote on the final core set of outcomes; this core set of outcomes consists of the outcomes that were initially included and excluded from the 2 rounds of the Delphi survey. At this stage, we will invite members from systematic review groups, clinical guideline developers, health care workers, and journal editors; we will also invite patients and caregivers to participate in the meeting. The inclusion criteria for health professionals are shown in Textbox 3. There are no exclusion criteria.

**The inclusion criteria for health professionals in the meeting.**

**Inclusion criteria**

- Health professionals with a bachelor’s degree or above.
- Health professionals who have a senior title or above.
- Clinicians and nurses should have work experience in tertiary hospitals.
- Researchers who have conducted systematic reviews of prostate cancer in the past 10 years.
- Journal editors should have at least 3 years of work experience.
- Policy makers should have at least 5 years of work experience.
- There will be no restriction on the professionals’ geographical area.

#### Sampling

Based on the published literature, there is no clear calculation method for the number of participants in consensus meetings [20]. However, to gather input from different stakeholders and improve consensus, we need to invite 20 professionals and 2-3 patients to participate.

#### Consensus Meeting Process

Participants who have participated in the 2 rounds of the Delphi survey will be requested to attend a web-based questionnaire meeting. In the consensus meeting, we will report the results of round 2 of the Delphi survey for health professionals and the results of the survey for patients. The outcomes labeled as “consensus out” by all stakeholders will be excluded. The outcomes labeled “consensus in” by all stakeholders will be sent to the Steering Committee and participants on the day preceding the consensus meeting.

In the consensus meeting, if participants disagree with an outcome that has achieved “consensus in” by all stakeholders to be included in the COS, they will further discuss it. Outcomes that do not reach a consensus will be individually addressed. Then, all participants in the consensus meeting will be anonymously asked to vote on these outcomes as “controversial” or “no consensus” outcomes. Any outcomes that receive votes from ≥ 70% of the participants will be included in the final COS.

## Results

The protocol has been registered in PROSPERO; we will initiate the review on August 1, 2023, and results should be expected by September 1, 2023. At the time of the revised submission, the systematic reviews have been completed. The Delphi survey among stakeholders is expected to start in October 2023. We will publish our results through TCM-related associations and the COMET platform to promote recognition and application of the core indicator set in a wider range.

The finished COS will follow the recommendation of recent studies on dissemination strategies [30-32]. Each participant of the COS will be asked to implement the COS in their future clinical trials about the application of TCM in prostate cancer treatment and recommend this COS to their colleagues and other potential researchers. We will communicate our results via peer-reviewed publications, conference presentations, professional societies, and our institution’s social media platforms. We will post our final COS information on the COMET website.

## Discussion

Constructing a core indicator set for the treatment of prostate cancer with TCM can improve the clarity of clinical research outcomes, thereby reducing the risk of publication bias and facilitating horizontal comparisons between different research results.

Prostate cancer is one of the most common malignant tumors affecting male individuals worldwide, posing a threat to patients’ health and quality of life [1,33]. However, currently published clinical trials focusing on TCM treatment for prostate cancer have encountered problems related to inconsistent and nonstandard reporting of outcome indicators [11-13].

Of note, there are certain limitations to this study. For instance, due to literature search strategies or language restrictions, it is possible that not all relevant clinical trials have been included in our analysis. Additionally, although we strive to fully adopt the views of multiple stakeholder groups, some biases may still be present.

This protocol design establishes a comprehensive review of both international and Chinese literature by conducting semistructured interviews, Delphi surveys, and a consensus meeting to fully adopt the views of multiple stakeholder groups, which can ensure the feasibility and promotion of the COS in future clinical studies. The development of a COS ensures the consistency of reporting clinical study outcomes for prostate cancer in the future, assisting in reducing reporting bias. This allows for easier comparison and integration of results from different clinical trials in the future, improving the value of these clinical studies and minimizing the waste of research resources.

## Appendix

Multimedia Appendix 1 Search terms used in the literature review.

## Abbreviations

ADT: androgen deprivation therapy
COMET: Core Outcome Measures in Effectiveness Trials
COS: core outcome set
PRISMA: Preferred Reporting Items for Systematic Reviews and Meta-Analysis
TCM: traditional Chinese medicine

## Data Availability

The data sets generated and analyzed during this study are available from the corresponding author on reasonable request.
