# Supplementary material for: Developing a Core Outcome Set for Assessing Clinical Safety Outcomes of Prostate Cancer in Clinical Trials of Traditional Chinese Medicine: Protocol for a Mixed Methods Study
Source: JMIR Res Protoc. 2023 Aug 7;12:e46794. doi: 10.2196/46794 (PMC10442731; doi:10.2196/46794)
Supplement: Multimedia Appendix 1 [file resprot_v12i1e46794_app1.docx]

Developing a core outcome set for assessing clinical safety outcomes of prostate cancer in clinical trials of Traditional Chinese medicine: study protocol

**Supplementary Materials**

[Search Terms Used in the Literature 2](#_Toc534313443)

[Search in PubMed 2](#_Toc534313444)

[Search in Cocrhane 3](#_Toc534313445)

[Search in Embase 4](#_Toc534313445)

**The complete PubMed search strategy is summarized in**

| **Number** | **Search terms** |
| --- | --- |
| #1 | Prostatic Neoplasms .MeSH Terms |
| #2 | Prostate Neoplasms .ti, ab. |
| #3 | Prostate Cancer .ti, ab. |
| #4 | Prostatic Cancer .ti, ab. |
| #5 | PCa .ti, ab. |
| #6 | #1 OR #2 OR #3 OR #4 OR #5 |
| #7 | Medicine, Chinese Traditional .MeSH Terms |
| #8 | Zhong Yi Xue .ti, ab. |
| #9 | Chung I Hsueh .ti, ab. |
| #10 | Decoction .ti, ab. |
| #11 | Capsule .ti, ab. |
| #12 | Pill .ti, ab. |
| #13 | Navel-applied .ti, ab. |
| #14 | #6 OR #8 OR #9 OR #10 OR #11 OR #12 OR #13 |
| #15 | Random* .ti, ab. |
| #16 | Clinical trial .ti, ab. |
| #17 | OR #15-16 |
| #18 | #6 AND #14 AND #17 |

**The complete Cocrhane search strategy is summarized in**

| **Number** | **Search terms** |
| --- | --- |
| #1 | MeSH descriptor [Prostatic Neoplasms] explode all trees |
| #2 | (Prostate Neoplasms):ti,ab,kw |
| #3 | (Prostate Cancer):ti,ab,kw |
| #4 | (Prostatic Cancer):ti,ab,kw |
| #5 | (PCa):ti,ab,kw |
| #6 | #1 OR #2 OR #3 OR #4 OR #5 |
| #7 | MeSH descriptor [Medicine, Chinese Traditional] explode all trees |
| #8 | (Zhong Yi Xue):ti,ab,kw |
| #9 | (Chung I Hsueh):ti,ab,kw |
| #10 | (Decoction):ti,ab,kw |
| #11 | (Capsule):ti,ab,kw |
| #12 | (Pill):ti,ab,kw |
| #13 | (Navel-applied):ti,ab,kw |
| #14 | #6 OR #8 OR #9 OR #10 OR #11 OR #12 OR #13 |
| #15 | (Random*):ti,ab,kw |
| #16 | (Clinical trial):ti,ab,kw |
| #17 | #15 OR #16 |
| #18 | #6 AND #14 AND #17 |

**The complete Embase search strategy is summarized in**

| **Number** | **Search terms** |
| --- | --- |
| #1 | Prostatic Neoplasms .exp |
| #2 | Prostate Neoplasms .ti,ab,kw |
| #3 | Prostate Cancer .ti,ab,kw |
| #4 | Prostatic Cancer .ti,ab,kw |
| #5 | PCa .ti,ab,kw |
| #6 | #1 OR #2 OR #3 OR #4 OR #5 |
| #7 | Medicine, Chinese Traditional .exp |
| #8 | Zhong Yi Xue .ti,ab,kw |
| #9 | Chung I Hsueh .ti,ab,kw |
| #10 | Decoction .ti,ab,kw |
| #11 | Capsule .ti,ab,kw |
| #12 | Pill .ti,ab,kw |
| #13 | Navel-applied .ti,ab,kw |
| #14 | #6 OR #8 OR #9 OR #10 OR #11 OR #12 OR #13 |
| #15 | Random* .ti,ab,kw |
| #16 | Clinical trial .ti,ab,kw |
| #17 | #15 OR #16 |
| #18 | #6 AND #14 AND #17 |
